# Luteolin Pretreatment Attenuates Hepatic Ischemia-Reperfusion Injury in Mice by Inhibiting Inflammation, Autophagy, and Apoptosis via the ERK/PPAR*α* Pathway

**DOI:** 10.1155/2022/8161946

**Published:** 2022-08-03

**Authors:** Yuhui Jiang, Wenjuan Yang, Jiameng Ding, Jie Ji, Liwei Wu, Yuanyuan Zheng, Yan Li, Ziqi Cheng, Jie Zhang, Qiang Yu, Jiao Feng, Jingjing Li, Jianye Wu, Yingqun Zhou, Chuanyong Guo

**Affiliations:** ^1^Department of Gastroenterology, Shanghai Tenth People's Hospital, Tongji University School of Medicine, Shanghai 200072, China; ^2^Department of Emergency, Shanghai Tenth People's Hospital, School of Medicine, Shanghai 200072, China; ^3^Department of Gastroenterology, Putuo People's Hospital, Tongji University, Shanghai 200060, China

## Abstract

Hepatic ischemia-reperfusion (IR) injury is a clinically significant process that frequently occurs in liver transplantation, partial hepatectomy, and hemorrhagic shock. The aim of this study was to explore the effectiveness of luteolin in hepatic IR injury and the underlying mechanism. BALB/c mice were randomly divided into six groups, including normal controls (NC), luteolin (50 mg/kg), sham procedure, IR+25 mg/kg luteolin, and IR+50 mg/kg luteolin group. Serum and tissue samples were collected at 6 and 24 h after reperfusion to assay liver enzymes, inflammatory factors, expression of proteins associated with apoptosis and autophagy, and factors associated with the extracellular signal-regulated kinase/peroxisome proliferator-activated receptor alpha (ERK/PPAR*α*) pathway. Luteolin preconditioning decreased hepatocyte injury caused by ischemia-reperfusion, downregulated inflammatory factors, and inhibited apoptosis and autophagy. Luteolin also inhibited ERK phosphorylation and activated PPAR*α*.

## 1. Introduction

Ischemia-reperfusion injury is caused by ischemic injury, followed by blood perfusion recovery, which affects all oxygen-dependent cells that depend on an uninterrupted blood supply. Tissues and organs are potential targets of ischemia-reperfusion injury because they include aerobic cells that depend on mitochondrial oxidative phosphorylation for energy [1, 2]. The liver is an oxygen-sensitive organ, and it may set off a severe chain reaction following IR injury [3, 4]. Hepatic ischemia-reperfusion injury (HIRI) is an exogenous antigen-independent local inflammatory response induced by hypoxic stress [5]. Energy metabolism, inflammatory responses, and various forms of cell death are critical processes involved in HIRI [6, 7]. Decreased cellular energy metabolism and increased oxidative stress are key responses in ischemia and the initial reperfusion phase. Mitochondrial function is impaired by free radicals and oxidants, and the generation of excess reactive oxygen species (ROS) induces subsequent inflammatory reactions. Inflammation is a critical event in both the initial and later phases of HIRI, in which the activation of Kupffer cells and neutrophils plays a central role [6]. Thus, inhibition of the augmented inflammatory response may prove to be an effective treatment of HIRI.

In addition to generating ROS and inducing inflammation, the activation of Kupffer cells and macrophages in HIRI can initiate apoptosis and autophagy [8, 9]. Apoptosis, or type1 programmed cell death, is highly regulated by two the BCL-2 family and the caspase family [10], which mediate hepatocyte death in the initial 24 hours after HIRI. Autophagy, whose role in liver diseases is still under debate, is regulated by autophagy-related (ATG) proteins including P62, Beclin-1, and LC3 [11]. Some researchers consider it to be a self-digestive process that maintains cell homeostasis and ensures cell survival under stressful conditions, while others consider it as type2 cell death, which is different from apoptosis with the accumulation of autophagosomes and autolysosomes in the cytoplasm [12]. Normally, ROS, apoptosis, and autophagy are all in dynamic equilibrium. However, after ischemia-reperfusion injury, the disturbed equilibrium will lead to cytotoxicity, hepatocyte dysfunction, and cell death [13], which also affects amounts of signaling pathways such as extracellular signal-regulated kinase (ERK) pathway and transcription factors such as PPAR*α* in hepatocytes.

Peroxisome proliferator-activated receptors (PPARs) are nuclear hormone receptors that comprise a superfamily of ligand-activated transcription factors. PPAR*α* is involved in lipid and lipoprotein metabolism, apoptosis, and inflammatory responses [14–16]. PPAR*α* activation relieves IR-induced liver, heart, and brain injury by suppressing inflammation, apoptosis, autophagy, and lipid peroxidation [17, 18]. ERK is a mitogen-activated protein kinase (MAPK) that is involved in the regulation cell proliferation, differentiation, apoptosis, survival, inflammation, and innate immunity [19–21]. The inhibition of ERK has positive effects on inflammation, apoptosis, autophagy, and other activities involved in HIRI [22–24]. There is evidence that ERK can regulate PPAR*α* activation in postprandial hepatic lipid metabolism [25], indicating that the interactions between ERK, PPAR*α*, and HIRI deserve to be investigated.

Luteolin is a flavone present in many vegetables, fruits, and medicinal herbs, and it has antioxidant, antimicrobial, anti-inflammatory, chemopreventive, chemotherapeutic, cardioprotective, antidiabetic, neuroprotective, and antiallergic activities [26–29]. Previous studies have shown that luteolin prevents liver injury caused by drugs or chemicals by suppressing inflammation, ROS, and autophagy [30–32]. Numerous studies also showed that the mechanism of luteolin on liver protection involves ERK inactivation and the consequent inhibition of apoptosis [33]. However, the exact benefits of luteolin on HIRI have not been described. The aim of this study was to investigate the hepatoprotective effectiveness of luteolin in HIRI and the underlying mechanisms. We hypothesized that luteolin could attenuate inflammation, inhibit apoptosis, and mediate autophagy via ERK/PPAR*α* pathway.

## 2. Materials and Methods

### 2.1. Reagents

Luteolin was purchased from Yuanye Biotechnology (Shanghai, China), dissolved in DMSO, and diluted in saline to the final concentration. Dimethyl sulfoxide (DMSO) was purchased from Sigma-Aldrich (St. Louis, MO, USA). Platelet-activating factor C-16 (PAF C-16) was purchased from Santa Cruz Biotechnology (Dallas, TX, USA). Alanine aminotransferase (ALT) and aspartate aminotransferase (AST) reagent kits were obtained from Jiancheng Bioengineering Institute (Nanjing, China). Quantitative real-time PCR kits were purchased from TaKaRa (Dalian, China). Detailed information on the primary antibodies used in our study is listed in Table 1. The anti-mouse and anti-rabbit secondary antibodies for western blot were purchased from LI-COR Biosciences (NE, USA). The secondary antibodies for immunohistochemistry were obtained from Servicebio (Wuhan, China). TdT-mediated dUTP nick end labeling (TUNEL) apoptosis assay kits were from Roche (Roche Ltd., Basel, Switzerland).

### 2.2. Animals

Male BALB/c mice weighing 21–25 g and 6–8 weeks of age were supplied by Shanghai SLAC Laboratory Animal Co., Ltd. (Shanghai, China). The mice were housed in plastic cages in a temperature-controlled environment at 22°C and an alternating 12 h:12 h light-dark circadian rhythm, with free access to food and water. All animal experiments were in compliance with the guidelines of the National Institutes of Health and were approved by the Animal Care and Use Committee of Tongji University in Shanghai.

### 2.3. Experimental Design

Sixty mice were randomly assigned to six treatments: (1) normal control group (NC, *n* = 6): mice were injected with vehicle intraperitoneally. (2) Sham group (*n* = 12): a laparotomy was performed, and the abdominal cavity was closed without IR injury. (3) Luteolin (50 mg/kg) group (*n* = 6): luteolin (50 mg/kg) was injected intraperitoneally once daily for 7 days. (4) IR group (*n* = 12): mice received HIRI surgery. (5) IR+luteolin (25 mg/kg) group (*n* =12): mice were injected intraperitoneally with 25 mg/kg luteolin once daily for 7 days before HIRI surgery. (6) IR+luteolin (50 mg/kg) group (*n* = 12): mice were injected with 50 mg/kg luteolin intraperitoneally once daily for 7 days before HIRI surgery. The doses were selected according to preliminary study [34]. Surgical procedures were done under anesthesia. Six mice in the sham group, IR group, IR+luteolin (25 mg/kg), and IR+luteolin (50 mg/kg) were randomly sacrificed 6 and 24 hours [35, 36] after reperfusion to collect blood and liver tissue for subsequent procedures.

### 2.4. Establishment of the HIRI Model

A model of segmental (70%) warm IR was established in mice after fasting for about 12 h with free access to water. Mice were anesthetized by intraperitoneal injection of 1.25% sodium pentobarbital (Nembutal; St. Louis, MO, USA). When the pain response disappeared, the mice were placed on a sterile table with their limbs immobilized. After disinfecting the abdominal skin, a midline laparotomy was performed to expose the liver hilum. The liver was carefully turned over to fully expose the first hepatic hilum. The anatomical structure of the hilum was carefully separated, and the blood vessels in the left and middle lobe of the liver were blocked with a sterile microvascular clamp for 45 minutes to immediately cause partial ischemia of the liver. The mice were placed on an electric blanket, and the incision was covered with wet saline gauze to maintain body temperature. After 45 minutes of ischemia, the clamp was removed, and the abdominal incision was sutured.

### 2.5. Cell Culture and Vitality

Normal hepatocyte LO2 cells were cultured in RPMI-1640 medium supplemented with 10% FBS, 100 U/mL of penicillin, and 100 mg/mL of streptomycin in a humidified incubator at 37°C with 5% CO_2_ and plated at a density of 2 × 10^4^ cells/well in 96-well plates in 100 *μ*L of medium per well. The cells were pretreated with luteolin in different concentrations (2.5 *μ*M, 5 *μ*M, 10 *μ*M, 20 *μ*M, and 40 *μ*M) for 24 hours. Then, the cells were administered with AR, which means hypoxia (3% O_2_, 5% CO_2_, and 92% N_2_) for 24 h and reoxygenation (5% CO_2_, 95% air) for 2 h, to simulate the process of HIRI in vitro. Cell viability was evaluated by CCK-8 assay.

LO2 cells were administered with or without 10 *μ*M luteolin and 4 *μ*M PAF C-16 according to the experiment design in the following five groups: (1) normal control (NC): no treatment; (2) AR+PAF C-16 group: 4 *μ*M PAF C-16 treated 24 h before IR; (3) AR group: administered with AR injury; (4) AR+luteolin group: 10 *μ*M luteolin preconditioned 24 h before AR; and (5) AR+PAF C-16+luteolin group: 10 *μ*M luteolin and 4 *μ*M PAF C-16 administered 24 h before AR.

### 2.6. Serum Assays

After storing blood samples at 4°C for 4–5 h, serum was obtained by centrifuging at 3500 rpm for 10 min. Serum levels of alanine transaminase (ALT) and aspartate aminotransferase (AST) were measured with microplate test kits.

### 2.7. Histopathology

The liver specimens removed from the left lobe were dehydrated in ethanol and embedded in paraffin. The specimens were cut into 3 *μ*m thick sections and stained with hematoxylin and eosin (H&E) to observe the degree of injury.

### 2.8. Immunohistochemistry

Paraffin sections of liver tissue were dewaxed in xylene and dehydrated in an alcohol gradient. Antigen retrieval was performed by incubating slides in citrate buffer in a 95°C water bath for 10 minutes, and the sections were soaked in 3% hydrogen peroxide for 10 minutes to block endogenous peroxidases. Sections were washed with PBS three times and treated with 5% bovine serum albumin (BSA) for 20 minutes to block nonspecific binding sites. The liver sections were then incubated overnight at 4°C with anti-tumor necrosis factor- (TNF-) *α*, anti-interleukin- (IL-) 1*β*, anti-IL-6, anti-Bcl-2, anti-Bax, anti-Beclin-1, anti-P62, anti-LC3, anti-PPAR*α*, and anti-p-ERK primary antibodies (all at 1: 200). The slices were incubated with a secondary antibody (1 : 200) for 1 h at 37°C the next day. A diaminobenzidine kit was used to visualize antibody binding by light microscopy. The stained area was measured by Image-Pro Plus software (version 6.0).

### 2.9. Reverse Transcription Polymerase Chain Reaction (RT-PCR) and Quantitative RT-PCR (qRT-PCR)

Total RNA was extracted from liver tissues by TRIzol reagent and reverse-transcribed into cDNA by with a reverse transcription kit (TaKaRa Biotechnology, Japan). The mRNA expression was quantified by SYBR Premix EX Taq and a 7900HT fast PCR system (Applied Biosystems, Foster City, CA, USA). The primer sequences used for PCR are listed in Table 2.

### 2.10. Western Blot Assays

Liver samples stored at −80°C and LO2 cells were lysed in radioimmunoprecipitation assay lysis buffer (Epizyme Biomedical Technology, Shanghai, China) containing protease inhibitors and phenylmethanesulfonyl fluoride, and the protein concentration was determined with bicinchoninic acid protein assay kits (Kaiji, China). Equal amounts of total protein were separated by 10% or 12.5% sodium dodecyl sulfate–polyacrylamide gel electrophoresis (SDS–PAGE) after being boiled at 100°C for 10 minutes and transferred onto 0.22 *μ*m polyvinylidene fluoride membranes. The membranes were blocked with 5% BSA or 5% nonfat milk for at least 1 h. After incubation overnight at 4°C with primary antibodies (Table 1), the membranes were washed three times with phosphate buffered saline with Tween (PBST) and then incubated with anti-rabbit or anti-mouse secondary antibodies for 1 h at 37°C. Protein expression was read with an Odyssey two-color infrared laser imaging system (LI-COR Biosciences, Lincoln, NE, USA) after washing the membranes three times in PBST. The gray values were quantified by ImageJ analysis software.

### 2.11. TUNEL Staining

After dewaxing and dehydration, liver tissue sections were digested in 20 *μ*g/mL proteinase K for 30 minutes and washed four times with PBS. The sections were incubated with TUNEL reaction buffer, and positively stained areas were observed by light microscopy.

### 2.12. Statistical Analysis

Results were reported as mean ± SD. All assays were performed at least three times. Differences among groups were analyzed by one-way analysis of variance with *p* < 0.05 considered statistically significant. Graphics were drawn with GraphPad Prism 8 software.

## 3. Results

### 3.1. Luteolin and Laparotomy Did Not Affect Liver Structure and Function

**Figure 1:**
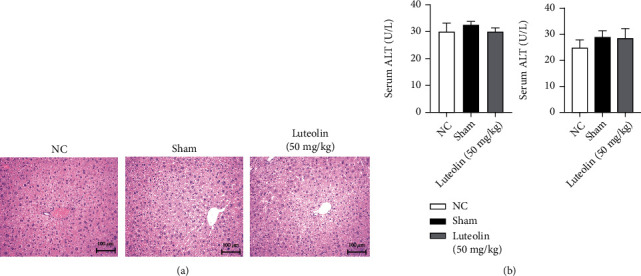
Effects of luteolin (50 mg/kg) and laparotomy on normal liver tissues. (a) Representative H&E staining in sections of the liver (original magnification, ×200). (b) Serum ALT and AST levels are presented as mean ± SD (*n* = 6, *p* > 0.05).

To verify that luteolin has no hepatotoxicity and does no harm to liver structures, we performed H&E staining and measured the ALT and AST levels of the NC group, sham group, and luteolin (50 mg/kg) group. H&E-stained sections of each group revealed no obvious changes (Figure 1(a)). There were no significant differences in the serum levels of the liver enzymes among the three groups (Figure 1(b)). Therefore, we believed that laparotomy and the high dose of luteolin did not affect liver structure and function.

### 3.2. Luteolin Preconditioning Mitigated Liver Function Injury Induced by HIRI

**Figure 2:**
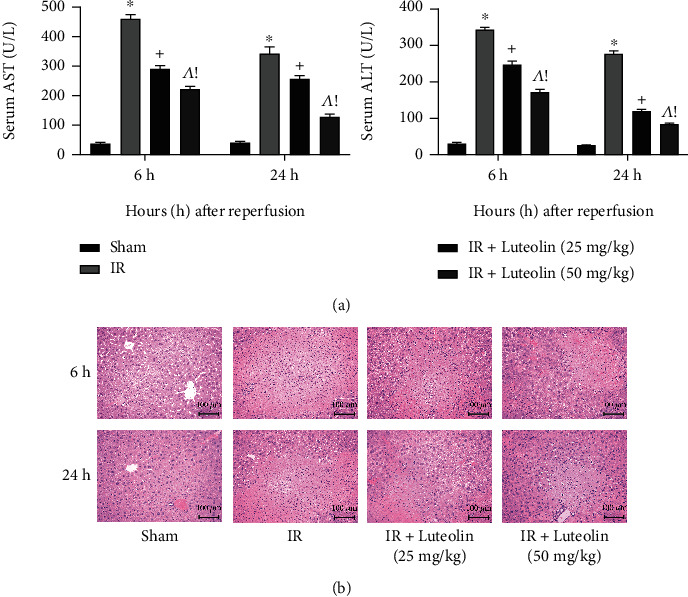
Effects of luteolin on the liver function and histopathology of hepatic IR mice. (a) The levels of serum ALT and AST are presented as mean ± SD (*n* = 6; ^∗^*p* < 0.05 for IR vs. sham; ^+^*p* < 0.05 for IR+luteolin (25 mg/kg) vs. IR; ^^^*p* < 0.05 for IR+luteolin (50 mg/kg) vs. IR; ^!^*p* < 0.05 for IR+luteolin (50 mg/kg) vs. IR+luteolin (25 mg/kg)). (b) H&E-stained liver sections were examined under light microscopy (magnification, ×200).

As biomarkers of hepatic function, serum ALT and AST levels reflect the extent of liver damage. Compared with the sham group, ALT and AST significantly increased in the IR group at 6 and 24 hours, and the increase was significantly smaller after luteolin preconditioning. In addition, the decrease of serum transaminase with luteolin appeared to be dosage-dependent (Figure 2(a)). Evaluation of H&E staining and liver pathology confirmed that laparotomy did not result in tissue damage in the sham group, while tissues from the IR group revealed extensive changes in liver tissue structure changes such as hepatocyte ballooning, necrosis, destruction of lobules, and inflammatory cell infiltration 6 hours after reperfusion. Luteolin pretreatment alleviated the damage caused by IR, with better protection achieved with 50 mg/kg luteolin than with 25 mg/kg (Figure 2(b)). Taken together, the results indicate that we successfully established the HIRI model and that luteolin protected against liver injury in a dose-dependent manner.

### 3.3. Luteolin Pretreatment Inhibited the Release of Inflammatory Factors

**Figure 3:**
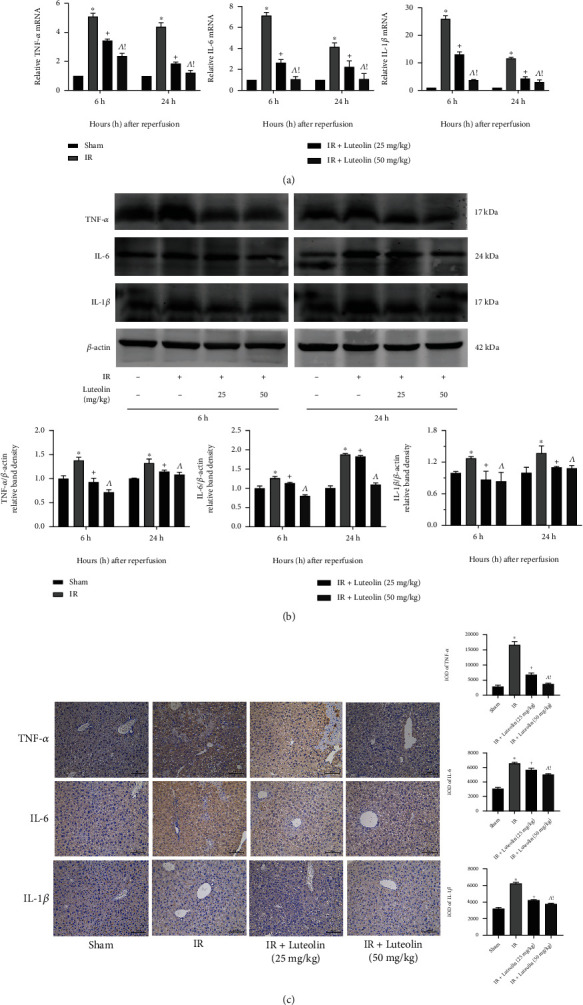
Luteolin reduced the expression of inflammatory cytokines. (a) Relative IL-6, IL-1*β*, and TNF-*α* mRNA levels were determined by qRT-PCR. (b) Western blot results of TNF-*α*, IL-6, and IL-1*β* protein levels. (c) Immunohistochemical staining (200x) showed expression of TNF-*α*, IL-6, and IL-1*β* proteins in liver tissues 6 hours after reperfusion. Final evaluations were made by Image-Pro Plus 6.0 software to calculate the IOD of the positive staining area. Data are presented as mean ± SD (*n* = 6; ^∗^*p* < 0.05 for IR vs. sham; ^+^*p* < 0.05 for IR+luteolin (25 mg/kg) vs. IR; ^^^*p* < 0.05 for IR+luteolin (50 mg/kg) vs. IR; ^!^*p* < 0.05 for IR+luteolin (50 mg/kg) vs. IR+luteolin (25 mg/kg)).

The release of inflammatory factors (e.g., TNF-*α*, IL-6, and IL-1*β*) after ischemia-induced macrophage activation is responsible for IR damage and for exacerbating liver microcirculation disorders. QRT-PCR, western blotting, and immunohistochemical (IHC) staining were used to explore the impact of luteolin on inflammation in mouse livers. The qRT-PCR results revealed that TNF-*α*, IL-1*β*, and IL-6 expressions were significantly higher in the IR group than in the sham groups and that luteolin preconditioning inhibited the release of the inflammatory mediators at both 6 and 24 h in a dose-dependent manner (Figure 3(a)). The western blotting results were consistent with those obtained by qRT-PCR (Figure 3(b)). IHC staining of liver sections showed that the expression of TNF-*α*, IL-6, and IL-1*β* was the highest in the IR group and that the expression of all three decreased with luteolin 25 mg/kg and 50 mg/kg pretreatment (Figure 3(c)). The results showed that luteolin attenuated liver inflammation in these mice with HIRI.

### 3.4. Luteolin Suppressed Apoptosis and Autophagy Induced by HIRI

**Figure 4:**
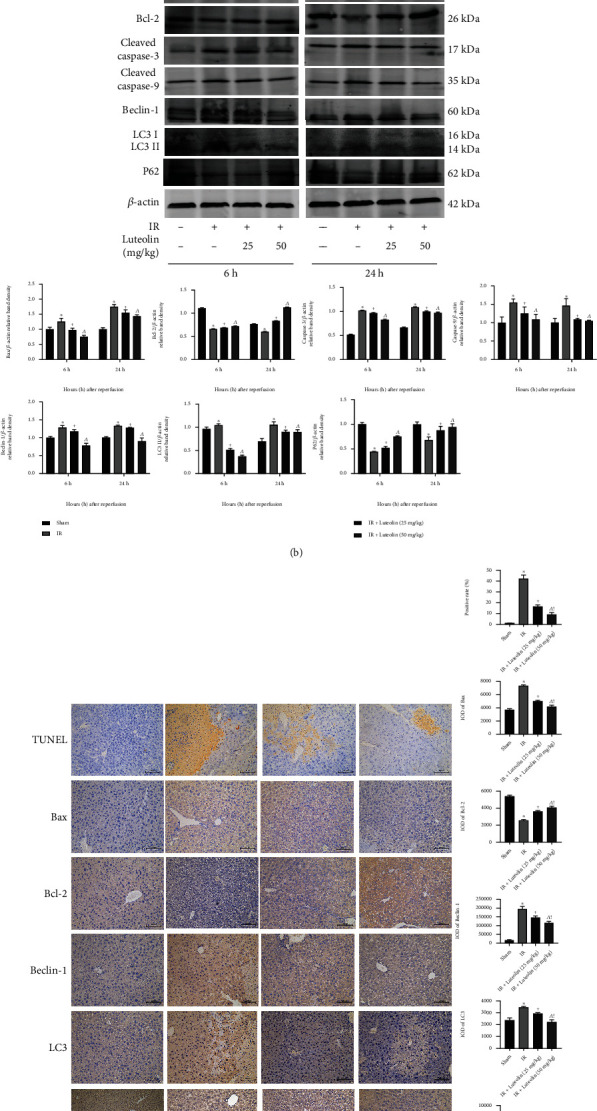
Luteolin attenuated IR-induced apoptosis and autophagy. (a) The relative mRNA levels of Bax, caspase 9, LC3, and p62. (b) Protein expression of apoptosis- and autophagy-related proteins. (c) After 6 h reperfusion, liver tissues were stained by TUNEL and observed under light microscopy (original magnification, ×200). Immunohistochemistry was used to detect Bcl-2, Bax, Beclin-1, LC3, and P62 expressions in liver tissues (original magnification, ×200). The IOD sum of brown area to total area was analyzed with the Image-Pro Plus software 6.0. Data were presented as mean ± SD (*n* = 6; ^∗^*p* < 0.05 for IR vs. sham; ^+^*p* < 0.05 for IR+luteolin (25 mg/kg) vs. IR; ^^^*p* < 0.05 for IR+luteolin (50 mg/kg) vs. IR; ^!^*p* < 0.05 for IR+luteolin (50 mg/kg) vs. IR+luteolin (25 mg/kg)).

Programmed cell death from apoptosis and autophagy both increases in HIRI injury. We assessed the extent of apoptosis by assaying Bcl-2, Bax, cleaved-caspase 3, and cleaved-caspase 9 expressions. The western blotting and qRT-PCR results indicated that both protein and mRNA expressions of Bax, caspase 3, and caspase 9 were significantly increased at 6 and 24 hours after reperfusion and that the increases were smaller with luteolin at 25 and 50 mg/kg in a dose-dependent manner (Figures 4(a) and 4(b)). The TUNEL assay and IHC results were consistent with those obtained by western blotting and qRT-PCR (Figure 4(c)). The expression of the antiapoptotic protein Bcl-2 was downregulated by IR surgery and upregulated by luteolin preconditioning, suggesting that luteolin protected the liver from IR injury by inhibiting apoptosis. Assays of the mRNA and protein expressions of autophagy-related factors Beclin-1, LC3, and P62 found that Beclin-1 and LC3 were upregulated in the IR model and were downregulated by luteolin administration. Changes in the expression of the antiautophagy protein P62 were in the opposite direction (Figures 4(a) and 4(b)). The results of IHC staining were consistent with those obtained with western blotting and qRT-PCR (Figure 4(c)). The results allow concluding that luteolin pretreatment inhibited activation of apoptosis and autophagy during HIRI.

### 3.5. Luteolin Suppressed ERK Phosphorylation and Activated PPAR*α*

**Figure 5:**
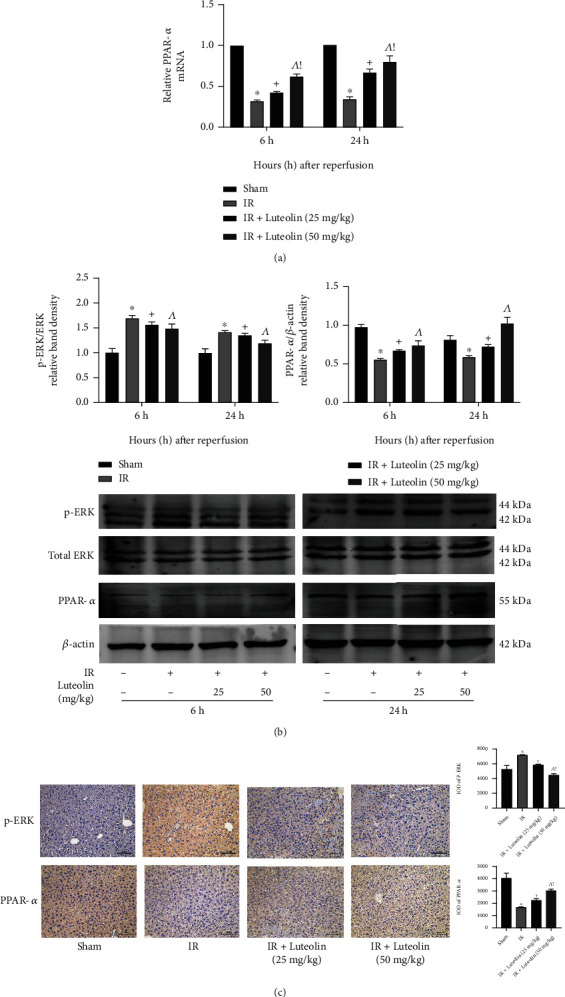
The protective effect of luteolin during hepatic IR injury is closely related with ERK/PPAR*α* pathway. (a) Relative PPAR*α* mRNA levels were determined by qRT-PCR. (b) Western blot results and analysis of total ERK, p-ERK, and PPAR*α* levels. (c) Levels of p-ERK and PPAR*α* in liver tissues at 6 hours after reperfusion are shown by immunohistochemical staining. Final evaluations were made by Image-Pro Plus 6.0 software to calculate the IOD of the positive staining area. Data are presented as mean ± SD (*n* = 6; ^∗^*p* < 0.05 for IR vs. sham; ^+^*p* < 0.05 for IR+luteolin (25 mg/kg) vs. IR; ^^^*p* < 0.05 for IR+luteolin (50 mg/kg) vs. IR; ^!^*p* < 0.05 for IR+luteolin (50 mg/kg) vs. IR+luteolin (25 mg/kg)).

Luteolin may protect against hepatocyte injury by reducing inflammatory responses and inhibiting apoptosis and autophagy. We tested the mechanism underlying the protective activity of luteolin by assaying ERK, phosphorylated ERK (p-ERK), and PPAR*α* expressions, all three of which participate in inflammation and programmed cell death. Differences in total ERK expression among the groups were not significant (Figure 5(b)), but differences in expression of p-ERK, which is the activated form of ERK, were significant. The results indicated that IR was associated with increased ERK phosphorylation and that luteolin preconditioning inhibited ERK activation (Figure 5(b)). The results are consistent with those obtained by IHC staining (Figure 5(c)). QRT-PCR, western blotting, and IHC staining revealed that PPAR*α* expression was downregulated in HIRI and that the downregulation was decreased by luteolin preconditioning in the IR groups (Figures 5(a), 5(b), and 5(c)). In short, luteolin protected the liver from IR injury by inhibiting ERK phosphorylation and activating PPAR*α*.

### 3.6. ERK/PPAR*α* Pathway Regulated Hepatocellular Apoptosis and Autophagy

**Figure 6:**
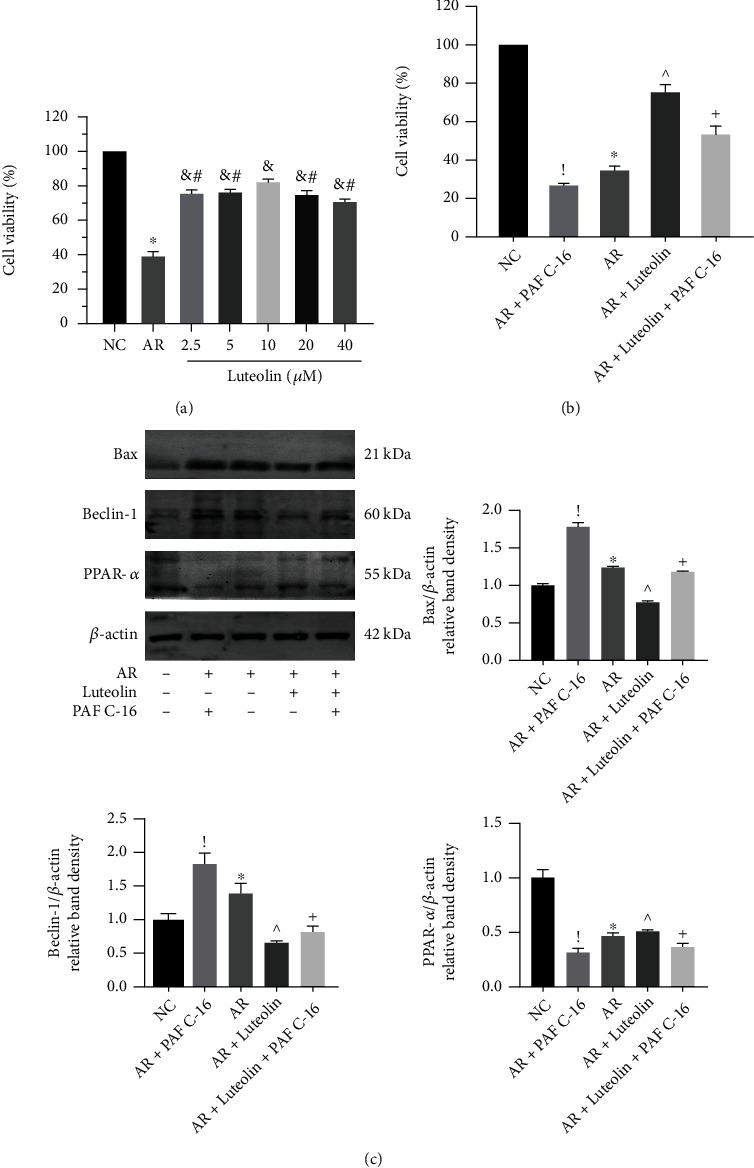
ERK/PPAR*α* pathway regulated hepatocellular apoptosis and autophagy. (a) Cell viability was measured by CCK-8 assay under different doses of luteolin (*n* = 3; ^∗^*p* < 0.05 vs. NC group; ^&^*p* < 0.05 vs. AR group; ^#^*p* < 0.05 vs. 10 *μ*M luteolin group). (b) Cell viability under different treatments was measured by CCK-8 assay. (c) Western blotting analysis of Bax, Beclin-1, and PPAR*α*. The western blot results were quantified with ImageJ 8.0 software (*n* = 3; ^!^*p* < 0.05 for AR+PAF C-16 vs. AR; ^∗^*p* < 0.05 for AR vs. NC; ^^^*p* < 0.05 for AR+luteolin vs. AR; ^+^*p* < 0.05 for AR+luteolin+PAF C-16 vs. AR+PAF C-16).

To further confirm the mechanism, we conducted in vitro experiments. Normal hepatocyte (LO2) cells were preconditioned with luteolin and administered with AR to stimulate HIRI injury in vitro. According to CCK-8 assay, 10 *μ*M luteolin was adopted for the following researches (Figure 6(a)). MAPK pathway agonist PAF C-16 was added to activate ERK/PPAR*α* pathway. The results revealed that the proportion of cell death was remarkably increased by IR treatment and the AR+PAF C-16 treatment, while luteolin can effectively protect hepatocytes from hypoxic injury and relieve the damage caused by ERK activation (Figure 6(b)). The expression of Bax and Beclin1 was detected by western blotting, indicating the degree of apoptosis and autophagy. Apparently, the expression was upregulated in AR group and in AR+PAF C-16 group whereas downregulated by luteolin administration. PPAR*α* expression was inhibited by AR and PAF C-16 treatment and recovered by luteolin pretreatment (Figure 6(c)). Thus, we deduced that luteolin ameliorated hepatocellular apoptosis and autophagy via ERK/PPAR*α* pathway.

## 4. Discussion

Hepatic IR injury is a pathophysiological process that occurs in numerous clinical settings, including hepatic injury, trauma, or shock. Previous studies have shown that liver IR injury not only leads to high mortality in patients undergoing liver surgery but also subsequently results in renal and myocardial injury [37–39]. Because of that, we attach special significance to the underlying mechanisms as well as possible strategies for the prevention and treatment of HIRI. Luteolin, a common flavonoid extracted from many edible plants [40], has been shown to protect against IR injury in the brain, heart, and kidney [41, 42]. Whether luteolin protects against HIRI is still unknown and is worth investigating. Therefore, we carried out this study to confirm the effects of luteolin on HIRI in mice.

Firstly, as a prerequisite for application, the nontoxicity of luteolin was confirmed by measuring the serum levels of ALT and AST in mice treated with 50 mg/kg luteolin compared with the normal control and sham groups. Changes in the structure of liver tissue structure were visualized by H&E staining. The above examinations revealed that 50 mg/kg luteolin was nontoxic in vivo. We then established a reliable model of HIRI in BALB/c mice with different doses of luteolin pretreatment. IR surgery resulted in substantial increases in serum ALT and AST, indicating extensive hepatocyte necrosis, but there was a dose-dependent decline in liver enzyme levels with luteolin preconditioning. Changes in the areas of necrotic tissue that were observed by light microscopy were consistent with changes in the ALT and AST levels, suggesting that the destruction of liver structures was alleviated by luteolin pretreatment.

The pathophysiology of HIRI is complicated, involving ATP depletion, mitochondrial permeability transition, imbalance of the endothelin/nitric oxide ratio, Ca_2_^+^ overload, macrophage activation, and other changes [43]. In the early stage of HIRI, hepatocytes under hypoxia and malnutrition decrease the production of adenosine triphosphate while increasing oxidative stress [44]. The huge increase of oxidative radicals results in inflammatory responses and activates Kupffer cells, endothelial cells, and other immune cells. Upon activation, the cells release more inflammatory factors, including IL-1*β*, IL-6, and TNF-*α*, which in turn intensify the release of ROS. Undoubtedly, the cascade reaction exacerbates ischemic injury [45]. Therefore, we used qRT-PCR, western blot, and IHC to investigate the expression of the cytokines IL-1*β*, IL-6, and TNF-*α* in the HIRI model. The results demonstrated that luteolin preconditioning attenuated the increases of IL-1*β*, IL-6, and TNF-*α* in a dose-dependent manner.

The activation of various immune cells and the subsequent excessive release of inflammatory factors induces autophagy and apoptosis in liver IR injury [6, 46]. The measurement of changes in Beclin-1, LC3, and P62 can help to explain how luteolin pretreatment regulates autophagy during HIRI as those proteins participate in the initiation of autophagy and the production of autophagosomes. P62 is thought an autophagy-specific substrate that interacts with multiple sites on the micro-tubule-associated protein light chain 3 (LC3), undergoes autooligomerization via the PB1 domain of p62, and enters the autophagy-lysosomal pathway to complete ubiquitination substrate degradation [47, 48]. Beclin-1 binds to Bcl-2 through its BH3-only domain to form a Beclin-1/Bcl-2 complex that is associated with the conversion of LC3 and may affect autophagy. In apoptosis process, activation of the proapoptotic protein Bax is followed by the formation of channels in the mitochondrial outer membrane and release of large proteins such as cytochrome c from the intermembrane space. Then, caspase 9 and caspase 3 are activated to induce apoptosis. Another member of Bcl-2 protein family, Bcl2, is an antiapoptosis mediator and inhibits the release of cytochrome c [49, 50]. Our results showed that luteolin preconditioning enabled the damaged liver to increase Bcl-2 and P62 expressions and decrease Beclin-1, LC3, Bax, caspase 9, and caspase 3 expressions. The qRT-PCR and IHC results are consistent with previous reports, indicating that luteolin attenuated autophagy and apoptosis during HIRI.

However, the exact molecular mechanisms are not clear and require further study. ERK is involved in cell development, colonization, apoptosis, and malignant transformation [51] that is activated by Raf serine/threonine kinases. Raf phosphorylates two serine residues on MEK1/2, resulting in the subsequent activation of ERK1/2. Phosphorylated ERK is active in various hepatic diseases such as liver fibrosis [52], nonalcoholic steatohepatitis [53], HIRI [24, 54], and hepatocellular carcinoma [55]. Studies of the impact of ERK on autophagy and apoptosis have been ongoing worldwide for many years. Inhibition of the RAF-MEK-ERK cascade was found to increase the dependence of pancreatic ductal adenocarcinoma on autophagy, thereby enhancing its responsiveness to autophagy inhibitors, suggesting a new treatment approach [56]. In inflammation-related diseases, ERK activation following the release of inflammatory mediators or ROS promotes apoptosis and autophagy, which leads to severe injury. Thus inhibiting ERK activation is a promising strategy for attenuating injury in inflammatory diseases.

PPARs are ligand-inducible transcription factors belonging to the nuclear receptor superfamily. PPAR*α* regulates lipid metabolism and mitochondrial function and may protect against liver injury by inhibiting inflammation [18, 57]. In response to inflammation, PPAR*α* activity depends on effector cells and transcription factors including nuclear factor kappa B (NF-*κ*B) and the signal transducers and activators of the transcription family [18, 58]. Previous studies have shown that increased expression of PPAR*α* alleviates inflammatory responses and regulates apoptosis and autophagy through AMP-activated protein kinase (AMPK), and the PI3K/AKT/mTOR signaling pathway in liver fibrosis and nonalcoholic fatty liver disease [59, 60], while the activity and influence of PPAR*α* on apoptosis and autophagy in HIRI are not well documented. Our study showed that luteolin can suppress autophagy and apoptosis caused by HIRI through inhibiting ERK activation and activating PPAR*α* in vivo, suggesting that both ERK and PPAR*α* participate in HIRI. However, the regulatory relationship between ERK and PPAR*α* is not yet clear. Researchers Jiao et al. have reported that PPAR*α* modulated the extent of inflammation by reducing MAPK phosphorylation [16], while Mooli et al. found that MEK-ERK signaling acts in postprandial hepatic lipid metabolism by regulating hepatic PPAR*α* signaling [25]. The differences indicate that more studies are needed. To further explore the mechanism, we carried out in vitro study. The western blot results revealed that PPAR*α* activation and expression were inhibited by AR injury and PAF C-16, the MAPK pathway agonist, whereas improved by luteolin treatment. The apoptosis and autophagy of hepatocytes were enhanced by AR and ERK activation while alleviated by drug treatment. So we can conclude that luteolin alleviates hepatocyte apoptosis and autophagy via ERK/PPAR*α* pathway.

**Figure 7:**
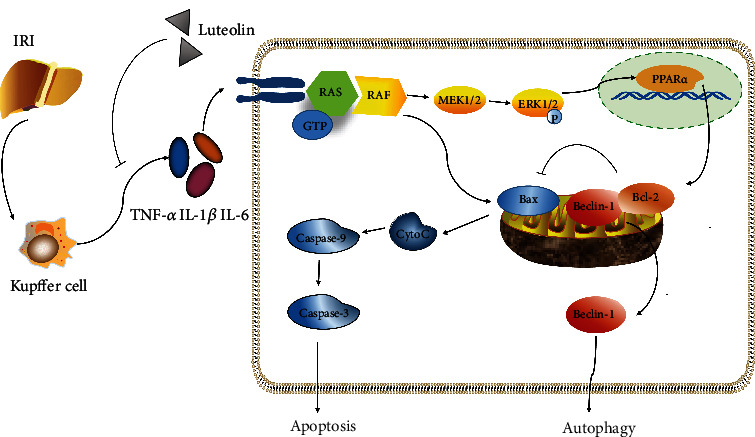
Probable mechanisms of luteolin against HIRI. Hepatic IR injury will activate Kupffer cells, thus producing several inflammatory cytokines, including IL-1*β*, IL-6, and TNF-*α*. Luteolin pretreatment can inhibit this process to alleviate liver injury. Luteolin can additionally regulate autophagy and apoptosis through ERK/PPAR*α* pathway.

In summary, luteolin had a pharmacological activity that protected the liver from damage caused by HIRI, and it involved the ERK/PPAR*α* pathway (Figure 7). The protective mechanisms included the elimination of inflammatory mediators and inhibition of autophagy and apoptosis. The results add to our understanding of hepatic IR injury but are limited because the relationship between luteolin and ERK/PPAR*α* pathway still needs further investigation, and the safety of luteolin for clinical use requires verification.

## 5. Conclusions

Luteolin attenuated HIRI in this mouse model. Luteolin decreased liver enzyme levels and alleviated pathological changes caused by HIRI. The hepatoprotective effects of luteolin were associated with inhibition of inflammation, autophagy, and apoptosis via the ERK/PPAR*α* pathway.

## Figures and Tables

**Table 1:** The primary antibodies used for western blotting in the study.

Antibody	Supplier	Catalogue number	Dilution	Molecular weight (kDa)
*β*-Actin	CST	3700	1 : 1000	42
TNF-*α*	CST	3707	1 : 1000	17
IL-1*β*	CST	12507	1 : 1000	17
IL-6	PT	21865-1-AP	1 : 1000	24
LC3	PT	14600-1-AP	1 : 2000	14.16
Beclin-1	PT	11306-1-AP	1 : 1000	60
Bcl-2	PT	26593	1 : 1000	26
Bax	Servicebio	GB11690	1 : 1000	21
Caspase 3	PT	19677-1-AP	1 : 1000	17
Caspase 9	PT	66169-1-Ig	1 : 1000	35
P62	PT	18420-1-AP	1 : 1000	62
PPAR*α*	PT	15540-1-AP	1 : 500	55
ERK	CST	4695	1 : 1000	42.44
P-ERK	CST	4370	1 : 2000	42.44

Abbreviations: PT: Proteintech (Chicago, IL, USA); CST: Cell Signaling Technology (Danvers, MA, USA).

**Table 2:** Oligonucleotide sequences of primers used for qRT-PCR.

Gene name	Forward (5′-3′)	Reverse (5′-3′)
*β*-Actin	GTGACGTTGACATCCGTAAAGA	GCCGGACTCATCGTACTCC
IL-6	CTGCAAGAGACTTCCATCCAG	AGTGGTATAGACAGGTCTGTTGG
IL-1*β*	GAAATGCCACCTTTTGACAGTG	TGGATGCTCTCATCAGGACAG
TNF-*α*	CAGGCGGTGCCTATGTCTC	CGATCACCCCGAAGTTCAGTAG
Bax	AGACAGGGGCCTTTTTGCTAC	AATTCGCCGGAGACACTCG
Caspase 9	GGCTGTTAAACCCCTAGACCA	TGACGGGTCCAGCTTCACTA
LC3	GACCGCTGTAAGGAGGTGC	AGAAGCCGAAGGTTTCTTGGG
P62	GAGGCACCCCGAAACATGG	ACTTATAGCGAGTTCCCACCA
PPAR*α*	AACATCGAGTGTCGAATATGTGG	CCGAATAGTTCGCCGAAAGAA

## Data Availability

The data used to support the findings of this study are available from the corresponding author upon request.
